# Can it be all more simple? Manufacturing aflatoxin biocontrol products using dry spores of atoxigenic isolates of *Aspergillus flavus* as active ingredients

**DOI:** 10.1111/1751-7915.13802

**Published:** 2021-03-23

**Authors:** Alejandro Ortega‐Beltran, Lawrence Kaptoge, Amadou L. Senghor, Morounranti O. S. Aikore, Patrick Jarju, Henry Momanyi, Matieyedou Konlambigue, Titilayo D. O. Falade, Ranajit Bandyopadhyay

**Affiliations:** ^1^ International Institute of Tropical Agriculture (IITA) Ibadan Nigeria; ^2^ IITA Dakar Senegal; ^3^ National Food Security, Processing and Marketing Corporation Banjul The Gambia; ^4^ IITA Nairobi Kenya; ^5^ IITA Accra Ghana

## Abstract

Aflatoxin contamination of staple crops, commonly occurring in warm areas, negatively impacts human and animal health, and hampers trade and economic development. The fungus *Aspergillus flavus* is the major aflatoxin producer. However, not all *A. flavus* genotypes produce aflatoxins. Effective aflatoxin control is achieved using biocontrol products containing spores of atoxigenic *A. flavus*. In Africa, various biocontrol products under the tradename Aflasafe are available. Private and public sector licensees manufacture Aflasafe using spores freshly produced in laboratories adjacent to their factories. BAMTAARE, the licensee in Senegal, had difficulties to obtain laboratory equipment during its first year of production. To overcome this, a process was developed in Ibadan, Nigeria, for producing high‐quality dry spores. Viability and stability of the dry spores were tested and conformed to set standards. In 2019, BAMTAARE manufactured Aflasafe SN01 using dry spores produced in Ibadan and sent via courier and 19 000 ha of groundnut and maize in Senegal and The Gambia were treated. Biocontrol manufactured with dry spores was as effective as biocontrol manufactured with freshly produced spores. Treated crops contained safe and significantly (*P* < 0.05) less aflatoxin than untreated crops. The dry spore innovation will make biocontrol manufacturing cost‐efficient in several African countries.

## Graphical abstract

Atoxigenic strain‐based aflatoxin biocontrol product Aflasafe is normally manufactured by coating carrier sorghum grains with a suspension of freshly produced spores of the active ingredients mixed with a polymer and a dye. We describe a process by which spores were produced and dried in Nigeria and transported to Senegal for manufacturing 190 tons of the biocontrol product. The effectiveness of the biocontrol formulation produced with dry spores and the conventional formulation were similar. The process with dry spores makes Aflasafe manufacturing efficient, versatile, and cost‐effective.

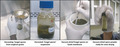

## Introduction

In tropical and sub‐tropical climates, fungi belonging to *Aspergillus* section Flavi frequently contaminate several crops with aflatoxins (Klich, 2007; Probst *et al*., 2014). Aflatoxins are highly toxic and carcinogenic compounds that negatively impact human and animal health, and hamper trade, income, and development outcomes in affected nations (Wu, 2015; Udomkun *et al*., 2017; JECFA, 2018). Throughout the world, the most common causal agent of aflatoxin contamination is *A. flavus* (Klich, 2007). However, there are *A. flavus* genotypes that naturally lack the ability to produce aflatoxins (from here on referred to as atoxigenic) due to defects in the aflatoxin biosynthesis gene cluster (Ehrlich and Cotty, 2004; Chang *et al*., 2005; Mehl *et al*., 2012). Some atoxigenic genotypes possess superior abilities to outcompete aflatoxin producers and are used as biocontrol agents to reduce crop aflatoxin content (Dorner, 2004; Cotty *et al*., 2007; Bandyopadhyay *et al*., 2016). Aflatoxin biocontrol products containing atoxigenic *A. flavus* genotypes as active ingredient fungi were first registered with the United States Environmental Protection Agency (USEPA) for use in cotton, groundnut, maize, pistachio, almond, and fig grown in the United States (Dorner, 2004; Cotty *et al*., 2007; Doster *et al*., 2014; Ortega‐Beltran *et al*., 2019).

Aflatoxin biocontrol products commercially used in the United States contain as active ingredient a single atoxigenic isolate belonging to a vegetative compatibility group (VCG) composed solely of atoxigenic members (Dorner, 2004; Mehl *et al*., 2012; Ortega‐Beltran and Bandyopadhyay, 2019). The International Institute of Tropical Agriculture (IITA), United States Department of Agriculture – Agricultural Research Service (USDA‐ARS), and several partners adapted and improved the biocontrol technology for use by smallholder farmers across sub‐Saharan Africa (SSA). Technology adaptation and improvement include (i) use of multiple atoxigenic isolates as active ingredient in a single product, and (ii) a manufacturing process adaptable to any country in SSA regardless of the context (Bandyopadhyay *et al*., 2016). Several biocontrol products under the tradename Aflasafe have been developed and registered for use in various countries in SSA. Each Aflasafe product contains, as active ingredient, a mixture of four isolates belonging to diverse atoxigenic African *Aspergillus flavus* VCGs (AAVs) native to the target country (Moral *et al*., 2020). The normal procedure to manufacture the biocontrol product is to multiply each active ingredient isolate in glass bottles containing sterilized sorghum grains, harvesting the spores in water to prepare a suspension, combining the spore suspension of each active ingredient in equal proportions, adding a polymer and a dye, and coating sterilized sorghum grains with the spore–polymer–dye suspension (Senghor *et al*., 2020).

A demonstration‐scale manufacturing plant was constructed at IITA‐Ibadan (Bandyopadhyay *et al*., 2016; Schreurs *et al*., 2019). The plant relies on receiving a freshly produced spore suspension of the active ingredients from a laboratory adjacent to the plant. IITA and partners constructed another manufacturing plant in Katumani, Kenya, to produce Aflasafe KE01, developed for use in Kenya, which also has its own spore production laboratory. Subsequently, a third plant was constructed in Kahone, Senegal, by BAMTAARE, a private company licensed to manufacture and distribute Aflasafe SN01, the product used in Senegal and The Gambia (Senghor *et al*., 2021). Aflasafe SN01 is registered with Le Comité Sahélien des Pesticides of Comité Inter‐Etate pour la Lutte contre la Sécheresse au Sahel (CSP/CILSS), the regulatory agency responsible for registering pesticides in 13 countries of the Sahel region. However, during its first year of planned operation, 2019, BAMTAARE had challenges to set up the spore production laboratory. BAMTAARE had a large order from a major groundnut processor that planned to work with a farmer cooperative to produce aflatoxin‐safe groundnut and there was the risk of failing to deliver the order.

With an unfinished spore production laboratory, manufacturing the product was not possible. To make available large amounts of spores, around 800 fragile bottles with spores from Ibadan, Nigeria, could have been sent via air courier to Senegal, but that practice is expensive, perilous, and could have resulted in poor‐quality active ingredients. Thus, to produce Aflasafe SN01 for use in the 2019 cropping season in Senegal and The Gambia, a process to dry the spores to a small volume was developed. The dry spores were sent via courier to Kahone, Senegal, where BAMTAARE staff combined them with the other ingredients at required rates and successfully manufactured the product to treat circa 19 000 ha of groundnut in Senegal and The Gambia.

We tested the hypothesis of whether biocontrol products manufactured using the conventional process and the new one differed in effectiveness to limit aflatoxin content in groundnut and maize grown in The Gambia and Senegal. The results of our investigations indicate that biocontrol was highly effective in limiting aflatoxin contamination regardless of whether dry spores or freshly produced spores were used to manufacture the product. Use of dry spores to manufacture biocontrol products in Senegal and elsewhere is poised to simplify the manufacturing process, ensure high‐quality products, reduce production costs, and consequently make the products cheaper to farmers.

## Results

### Viability and purity of dry spores

There were no differences in viability of the dry spores and the freshly harvested spores, regardless of isolate and storage period (data not shown). In addition, the drying process and storage period did not result in detectable contamination of the spores by other microbes.

### Spore production in products manufactured using dry spores and freshly harvested spores

After incubation, in all of the paired comparisons, biocontrol products manufactured with dry spores produced similar number of spores (*P* > 0.05) as products manufactured with freshly harvested spores. This occurred regardless of isolate evaluated and period for which the dry spores were stored (Table 1). In addition, storage period had no effect on the number of spores produced. For example, spore yield in products manufactured with M21‐11 dry spores stored for 2 and 30 days were 13.58 × 10^5^ spore grain^−1^ and 13.39 × 10^5^ spore grain^−1^, respectively (Table 1). Although not statistically significant, there were some cases in which lower or higher sporulation was detected in either dry spore‐ or fresh spore‐based products, but there were no consistent trends for specific isolates, storage period, or whether the spores were freshly harvested or dried.

**Table 1:** Spores produced per carrier grain of biocontrol product manually manufactured with spores of atoxigenic *Aspergillus flavus* isolates subjected to the vacuuming and drying process compared to product conventionally manufactured with freshly produced spores.

*A. flavus* isolate^a^	CFU/grain × 10^6^ ^b^, ^c^									
	Day 2		Day 5		Day 10		Day 15		Day 30	
	Dry	Fresh	Dry	Fresh	Dry	Fresh	Dry	Fresh	Dry	Fresh
M2‐7	132.9	140.8	119.8	131.2	119.0	116.6	121.2	138.8	111.2	136.1
M21‐11	135.8	130.9	113.3	128.8	116.4	131.5	135.8	105.9	133.9	115.1
MS14‐19	133.0	136.7	113.5	126.0	111.3	111.4	123.0	101.7	112.6	119.8
SS19‐14	124.9	136.1	116.1	115.1	117.4	119.8	120.0	122.2	112.9	122.1
Mean per treatment^d^	130.8AB, z	136.1A	115.7B, y	125.3AB	116.0B, y	119.8B	125.0AB, yz	117.1B	117.7B, yz	123.3AB
Mean per day^e^	133.5Z		120.5Y		117.9Y		121.0Y		120.5Y	
Overall mean^f^	122.7									

^a^ The four atoxigenic isolates of *A. flavus* are the active ingredient fungi of the aflatoxin biocontrol product Aflasafe SN01, registered for use in Senegal and The Gambia.

^b^ CFU: Colony Forming‐Units. CFU values were calculated using a turbidimeter (Agbetiameh *et al*., 2019).

^c^ In Day 2 values, Dry refers to CFU/grain of a product manually manufactured with 7‐d‐old spores filtered, dried for 48 h at 37°C, and stored for 2 days prior to manufacture (e.g. for M2‐7: 132.9 × 10^6^). Fresh (e.g. for M2‐7: 140.8 × 10^6^) refers to CFU/grain of a product made with spores harvested within 20 min prior to manufacture. The same rationale applies to other comparisons: products made with spores dried and stored for 5, 10, 15, or 30 days, and compared with products made conventionally with freshly harvested spores. Both types of products were incubated in a moist environment for 7 days at 31°C. Then, grains were shaken in water, and CFU per carrier grain calculated. None of the values in any of the individual atoxigenic isolate comparisons (i.e. Dry vs Fresh at each day) were significantly different from each other by Student’s *t*‐test (α = 0.05).

^d^ Mean CFU values of the four isolates in each day, in each treatment. Values with the same uppercase letters are not significantly different (Tukey’s HSD, α = 0.05). Values for Dry spore treatments were compared separately and those with the same lowercase letter are not significantly different (Tukey’s HSD, α = 0.05).

^e^ CFU values of the four isolates in both Dry and Fresh treatments, at the indicated date. Values with the same uppercase letters are not significantly different (Tukey’s HSD, α = 0.05).

^f^ Mean CFU/grain values of all isolates, in all treatments, at each day.

A more detailed analysis revealed some differences for a few of the evaluations. For example, there was significantly (*P* < 0.05) higher mean spore production of the combined four isolates in products manufactured with fresh spores in Day 2 than in those produced with dry spores stored for 5, 10, and 30 days but also in those conventionally produced with fresh spores in Day 10 and 15 (Table 2). The rest of the treatments formed an intermediate group between the lower and higher spore‐producing treatments. When combining values of the four isolates in products made with freshly produced and dry spores, higher (*P* < 0.05) spore production occurred on Day 2 compared other days. Similarly, a separate analysis for dry spore product revealed significantly higher (*P* < 0.05) spore production on Day 2. On the other hand, pooling all data together – regardless of isolate and days of storage – showed no differences (*P* > 0.05) in number of spores produced in products made with freshly produced and dry spores. Also, across days and treatments, all isolates had similar spore production values (*P* > 0.05).

**Table 2:** Aflatoxin content in groundnut sampled from (i) fields treated with the biocontrol product Aflasafe SN01 formulated either conventionally or with dry spores, and (ii) untreated fields in two regions of The Gambia during the 2019 cropping season.

Region	Formulation^a^	Treatment^b^	*n*	Total aflatoxin (ppb)^c^			Variance	Red (%)^e^
				Min	Max	Average^d^		
Central River	Dry spores	Treated	10	2.0	2.0	2.0b*	0.0	96.8
		Untreated	10	18.0	170.0	62.5a	1986.7	
	Conventional	Treated	10	2.0	2.0	2.0b**	0.0	95.5
		Untreated	10	25.0	70.0	44.0a	237.8	
North Bank	Dry spores	Treated	10	2.0	2.0	2.0b*	0.0	97.1
		Untreated	10	25.0	190.0	68.0a	2247.1	
	Conventional	Treated	10	1.0	2.0	1.9b*	0.1	95.7
		Untreated	10	25.0	100.0	44.0a	476.7	

^a^ Dry spore refers to product manufactured in Kahone, Senegal, in 2019 with a new process using dry spores produced in Ibadan, Nigeria. Conventional refers to product manufactured in Ibadan during 2018 using the standard process previously described (Bandyopadhyay *et al*., 2016). All farmers belong to the National Food Security, Processing and Marketing Corporation of The Gambia.

^b^ Treated refers to fields to which Aflasafe SN01, regardless of formulation, was applied at the rate of 10 kg ha^‐1^. Untreated were nearby fields in which no biocontrol product was applied and were separated by at least 200 m from corresponding treated field.

^c^ Aflatoxin values are in parts per billion (ppb).

^d^ Means of aflatoxin values were compared independently between treated and untreated crops in each formulation type and each region. Treated values with one (*) or two asterisks (**) significantly differed from corresponding untreated values by Student’s *t*‐test (α = 0.05 and 0.01, respectively). In addition, values in each treatment were compared regardless of region, treatment, and formulation. Values with the same lowercase letter are not significantly different (Kruskal–Wallis test, α = 0.05).

^e^ Percentage reduction was calculated as follows: ([mean of untreated − mean of Aflasafe SN01 treated]/mean of untreated) × 100.

### Quality test of biocontrol products manufactured manually and industrially using dry spores

All experimental products manufactured in Ibadan with dry spores yielded grains colonized exclusively by the atoxigenic *A. flavus* isolate that was inoculated. None of the grains of any of the experimental products contained any other microorganism.

Similarly, each batch of biocontrol product manufactured in Kahone with dry spores had carrier grains colonized exclusively by *A. flavus* and only fungi belonging to one of the four atoxigenic AAVs of Aflasafe SN01 were detected. None of the grains contained any other microorganism. Each atoxigenic AAV composed 25 ± 3% of the fungi coated on the carrier grains. The number of spores on each g of biocontrol product was, on average, 3500 ± 300.

### Aflatoxin levels in treated and untreated maize and groundnut grains

#### 2019 – Dry spore product vs. Conventional product in The Gambia

Total aflatoxin content in treated groundnut was never above 2 parts per billion (ppb), regardless of formulation used and region where the trials were conducted (Table 2). Both dry spore and conventional formulations were equally effective in limiting aflatoxin contamination (*P* < 0.05). In contrast, total aflatoxin content in untreated groundnut ranged from 18 to 190 ppb. The variance in treated crops was negligible (up to 0.1) while in untreated crops it ranged from 238 to 2247 (Table 2). The Kruskal–Wallis test revealed that all treated crops, regardless of formulation and region, had statistically similar aflatoxin levels but significantly less than those of untreated crops (*P* < 0.0001). Aflatoxin reductions in treated crops ranged from 95.5% to 97.1% (Table 2).

#### 2018 (conventional product) and 2019 (dry spore product)

In The Gambia, commercial use of conventional product by farmers belonging to the National Food Security, Processing and Marketing Corporation (NFSPMC) in 2018 resulted in total aflatoxin levels ranging from 1 to 4 ppb while untreated groundnut had from 2 to 170 ppb (Senghor *et al*., 2021; Table 3). Aflatoxin reductions in treated groundnut ranged from 82% (avg. aflatoxin level in corresponding untreated groundnut = 12.8 ppb) to 96% (avg. aflatoxin level in corresponding untreated groundnut = 53.1 ppb).

**Table 3:** Aflatoxin content in groundnut sampled from (i) fields treated with the biocontrol product Aflasafe SN01 formulated either conventionally (2018) or with dry spores (2019), and (ii) untreated fields in the Central River, North Bank, and West Coast regions of The Gambia.

Region	Farmers organization^a^	Year	Formulation^b^	Treatment^c^	*n*	Total aflatoxin ppb^d^				Red (%)^f^
						Min	Max	Average^e^	Variance	
Central River	NFSPMC	2018	Conventional	Treated	15	2.0	3.0	2.1g**	0.1	96.0
				Untreated	15	11.0	170.0	53.1de	1846.0	
	NFSPMC	2019	Dry spores	Treated	15	1.0	3.0	2.0g**	0.3	98.5
				Untreated	15	65.0	380.0	133.8a	6524.3	
North Bank	NFSPMC	2018	Conventional	Treated	15	1.0	3.0	2.3g**	0.4	82.0
				Untreated	15	2.0	30.0	12.8f	92.0	
	NFSPMC	2019	Dry spores	Treated	15	ND	2.0	1.1g**	0.5	98.0
				Untreated	15	14.0	120.0	53.1cde	690.1	
West Coast	NFSPMC	2018	Conventional	Treated	15	2.0	4.0	2.3g**	0.4	93.6
				Untreated	15	12.0	71.0	35.9e	292.0	
	NFSPMC	2019	Dry spores	Treated	15	ND	3.0	1.5g**	0.8	98.8
				Untreated	15	22.0	150.0	75.5bcd	1786.1	
Central River	FAO—Njao	2019	Dry spores	Treated	10	2.0	3.0	2.3g**	0.2	98.0
				Untreated	10	87.0	120.0	100.0ab	107.6	
	FAO—Kaur	2019	Dry spores	Treated	10	1.0	4.0	2.1g*	0.5	97.8
				Untreated	10	59.0	210.0	96.4abc	3167.8	
	FAO—Niani	2019	Dry spores	Treated	10	2.0	2.0	2.0g**	0.0	97.5
				Untreated	10	49.0	110.0	79.9abc	385.4	
	FAO—Sami	2019	Dry spores	Treated	10	2.0	2.0	2.0g**	0.0	97.9
				Untreated	10	77.0	120.0	93.2ab	183.3	

^a^ NFSPMC: National Food Security, Processing and Marketing Corporation of The Gambia. FAO: Food and Agriculture Organization. Farmers worked with FAO in four villages in Central River: Njao, Kaur, Niani and Sami.

^b^ Dry spore refers to product manufactured in Kahone, Senegal, in 2019 with a new process using dry spores produced in Ibadan, Nigeria. Conventional refers to product manufactured in Ibadan during 2018 using the standard process previously described (Bandyopadhyay *et al*., 2016).

^c^ Treated refers to fields to which Aflasafe SN01, regardless of formulation, was applied at the rate of 10 kg ha^‐1^. Untreated were nearby fields in which no biocontrol product was applied and were separated by at least 200 m from corresponding treated field.

^d^ Aflatoxin values are in parts per billion (ppb).

^e^ Means of aflatoxin values were compared independently between treated and untreated crops in each region, famers’ organization, and year. Treated values with one (*) or two asterisks (**) significantly differed from corresponding untreated values by Student’s *t*‐test (α = 0.01 and 0.001, respectively). In addition, values in each treatment were compared regardless of region, farmers organization, year, treatment, and formulation. Values with the same lowercase letter are not significantly different (Tukey’s HSD, α = 0.05).

^f^ Percentage reduction was calculated as follows: ([mean of untreated − mean of Aflasafe SN01 treated]/mean of untreated) × 100.

Commercial use of dry spore product by NFSPMC farmers in 2019 resulted in aflatoxin levels ranging from not detectable to 3 ppb (Table 3). On the other hand, the corresponding untreated groundnut had aflatoxin levels ranging from 14 to 380 ppb. Treated groundnut had 98% to 99% less aflatoxin than untreated groundnut. Treated groundnut by farmers participating in a Food and Agriculture Organization (FAO) scheme during 2019, also with dry spore product, had aflatoxin levels ranging from 1 to 4 ppb. Treated groundnut by FAO farmers had 97.5–98.0% less aflatoxin than untreated groundnut. Aflatoxin levels in all treated crops, regardless of formulation, year, region, and farmers’ organization were statistically similar but significantly different from untreated crops (*P* < 0.0001). In addition, there were significant (*P* < 0.0001) differences in aflatoxin content among untreated crops. Overall, untreated crops from 2018 contained less aflatoxin than those from 2019 (Table 2).

In Senegal, commercial use of conventional product by BAMTAARE farmers in 2018 on groundnut and maize resulted in 2.2–3.7 ppb total aflatoxin while in untreated crops aflatoxin levels ranged from 5.5 to 51.3 ppb (Table S1). Aflatoxin reductions in treated crops ranged from 55% (avg. aflatoxin level in corresponding untreated crop = 5.3 ppb) to 94% (avg. aflatoxin level in corresponding untreated crop = 37.8 ppb). Also in Senegal, but in 2019 and with dry spore product, crops treated by either BAMTAARE and ASPRODEB farmers had aflatoxin levels ranging from 2 to 3 ppb while corresponding untreated crops had aflatoxin levels ranging from 2.5 to 51.2 ppb (Table S1). Aflatoxin reductions in treated crops ranged from 15% (avg. aflatoxin level in corresponding untreated crop = 2.5 ppb) to 96% (avg. aflatoxin level in corresponding untreated crop = 51.2 ppb).

Regardless of country, product, crop, year, or farmer organization, variance was very stable in treated crops, always < 2 (except for 1 case in which the variance was 30.5). In contrast, the variance went up to 3402 and 6524 in untreated crops in Senegal and The Gambia, respectively (Table 3 and Table S1).

## Discussion

**Fig. 1:**
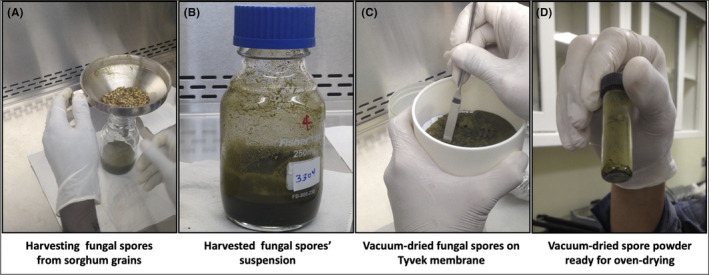
A brief description of the process to dry spores of atoxigenic isolates of *Aspergillus flavus*. Harvesting of fungal spores from colonized sorghum grains using sterile funnels with the stem covered with a sterile 1.7‐mm^2^ sieve (A). Harvested fungal spores in suspension in a 250 ml glass bottle (B). Vacuum‐dried fungal spores on Tyvek® membrane (lining the sieve in a Büchner funnel) being collected using a sterile spatula (C). Spore powder transferred to a sterile glass vial and ready for drying in the oven; note minimum humidity at the bottom of the vial (D).

The current study reports (i) the pathway to manufacture an aflatoxin biocontrol product in Senegal using a novel methodology for IITA and partners, and (ii) the effectiveness of the product in commercially grown crops in both Senegal and The Gambia. A newly constructed manufacturing facility in Kahone by the company BAMTAARE had an incomplete spore production laboratory. Thus, manufacturing the product Aflasafe SN01 for use during the 2019 cropping season was not feasible. To overcome this, the active ingredient spores were produced in Ibadan, Nigeria, and harvested into a dry powder (Fig. 1A–D). Laboratory tests showed both that the viability of the dry spores was unaffected and that products manufactured with dry spores yielded similar fungal reproduction as those manufactured with freshly harvested spores (Table 1). Products manufactured either with the conventional method or with dry spores were equally effective at limiting aflatoxin contamination in groundnut and maize commercially grown by smallholder farmers in The Gambia and Senegal. Our inferences are based on 2‐year results from 770 fields (385 treated, 385 untreated; Tables 2 and 3, Fig. 3). Most treated crops (98%) had total aflatoxin levels at or below 4 ppb (EU tolerance threshold). In contrast, untreated crops had up to 150 and 380 ppb total aflatoxin in Senegal and The Gambia, respectively.

Development, testing and registration of an aflatoxin biocontrol product containing atoxigenic isolates of *A. flavus* as active ingredients can take over a decade. Thereafter, several actions are needed to manufacture and distribute the product at scale (Ortega‐Beltran and Bandyopadhyay, 2019; Schreurs *et al*., 2019; Andrews *et al*., 2020; Konlambigue *et al*., 2020). First, a strategy must be developed to guide the commercialization process. Then, private sector companies evaluate the business case to invest in the technology. Interested companies are vetted to select the most suitable one to which the biocontrol technology is licensed for manufacturing and/or distribution. However, many factors can delay the construction, fine‐tuning, and commissioning of a manufacturing facility. Delivery of equipment to produce the spores and/or manufacture the biocontrol product *per se*, and/or to verify the quality of the finished product may be delayed for several reasons (e.g. shipping time, custom issues, and unexpected damage).

The first aflatoxin biocontrol product manufacturing plant – in Phoenix, Arizona – was set up with two laboratories, one for spore production and another for quality tests, and a manufacturing hall (Cotty *et al*., 2007). Similarly, the first two aflatoxin biocontrol manufacturing plants in Africa had the two laboratories and the manufacturing hall (Bandyopadhyay *et al*., 2016). On the other hand, the biocontrol product Afla‐Guard® is manufactured in the United States with spores produced in Japan by a third‐party service provider (Dorner, 2004). Therefore, IITA considered feasible seeking a cost‐effective method to produce dry spores to supply manufacturers of aflatoxin biocontrol products in African countries with active ingredient spores. In 2019, production of spores at Kahone was planned for manufacture of Aflasafe SN01 but the laboratory to produce them was not finished on time. A method to produce dry spores was thus rapidly developed using commonly available laboratory equipment (Fig. 1) so as to not lose the cropping season. We found that drying the spores had no effect on their viability compared to freshly harvested spores. There were some instances in which higher or lower spore production occurred in products made with dry spores but there were no consistent trends to associate lower spore production in either type of formulation (Table 1). In general, experimental products manufactured with dry spores stored for up to 30 days had similar fungal reproduction levels as products manufactured with freshly harvested spores when incubated for 7 days at 35°C (Table 1). Based on those results BAMTAARE decided to manufacture the product in Kahone with spores produced and dried in Ibadan.

**Fig. 2:**
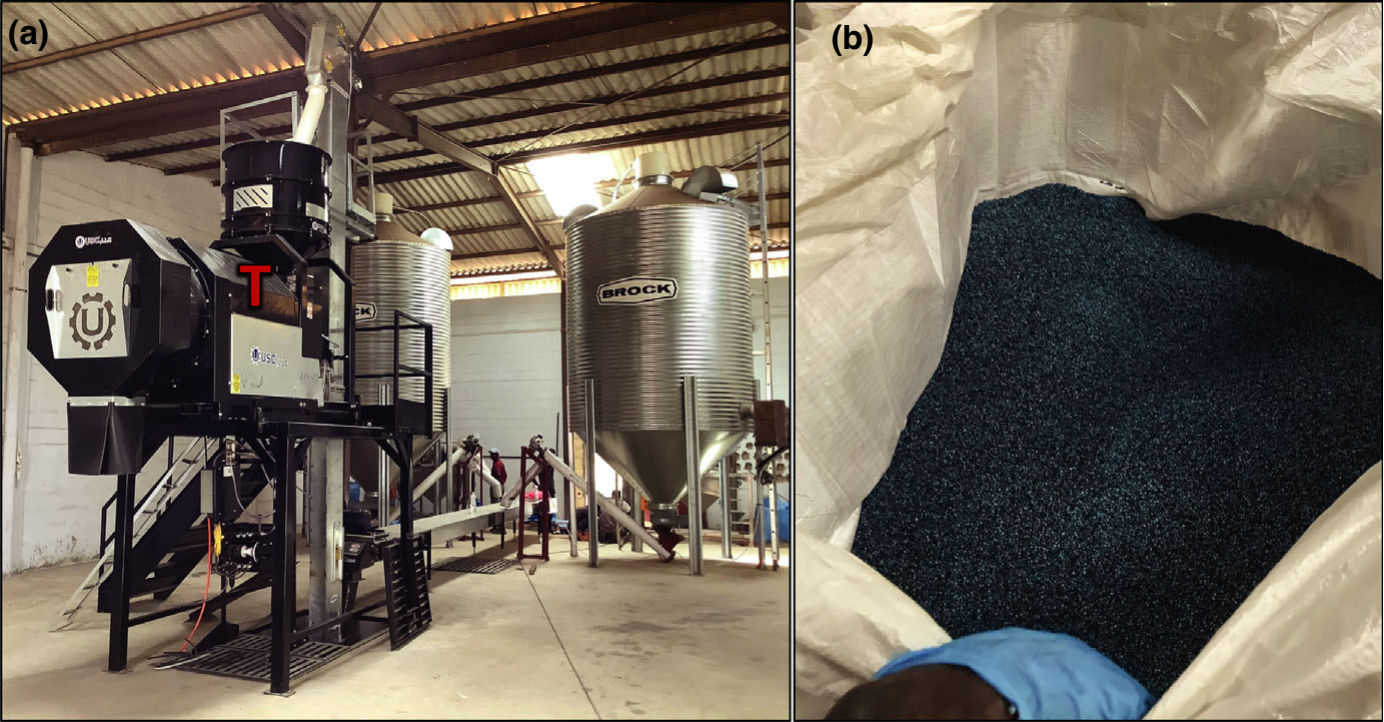
Manufacturing facility in Kahone, Senegal where a seed treater (T) is used to coat roasted sorghum grains with a mixture of spore suspension, blue food colorant and a polymer (A). Biocontrol product temporarily stored in a 1‐ton bag and ready to be packaged in 5 kg plastic bags (B).

In Kahone, appropriate quantities of dry spores (5 g from each of the four atoxigenic isolates of *A. flavus* composing Aflasafe SN01) were combined with required volumes of water, polymer, and blue dye to produce 1 ton of biocontrol product (Fig. 2). After coating, the product was packaged and stored. Quality tests of samples collected from each of the 360 batches produced (500 kg per batch) revealed that the product had same quality as that produced with freshly harvested spores: there was absence of contamination, only Aflasafe SN01 AAVs were present in the carrier grains, and appropriate number of viable spores were coated per g of product. Those tests were conducted in the Quality Test laboratory of the facility in Kahone, which was finished on time. A total of 190 tons of Aflasafe SN01 were manufactured using this process allowing treatment of 19 000 ha of groundnut and/or maize. After confirming its high quality, the product was bought by commercial farmer organizations in Senegal and The Gambia. In addition, 1 ton of the product was sent to Mali for research field effectiveness trials in maize, sorghum, and groundnut (unpublished results). It is important to note that the 4 kg of dry spores sent to Kahone are sufficient to produce 200 tons of the biocontrol product (although 190 tons were produced).

**Fig. 3:**
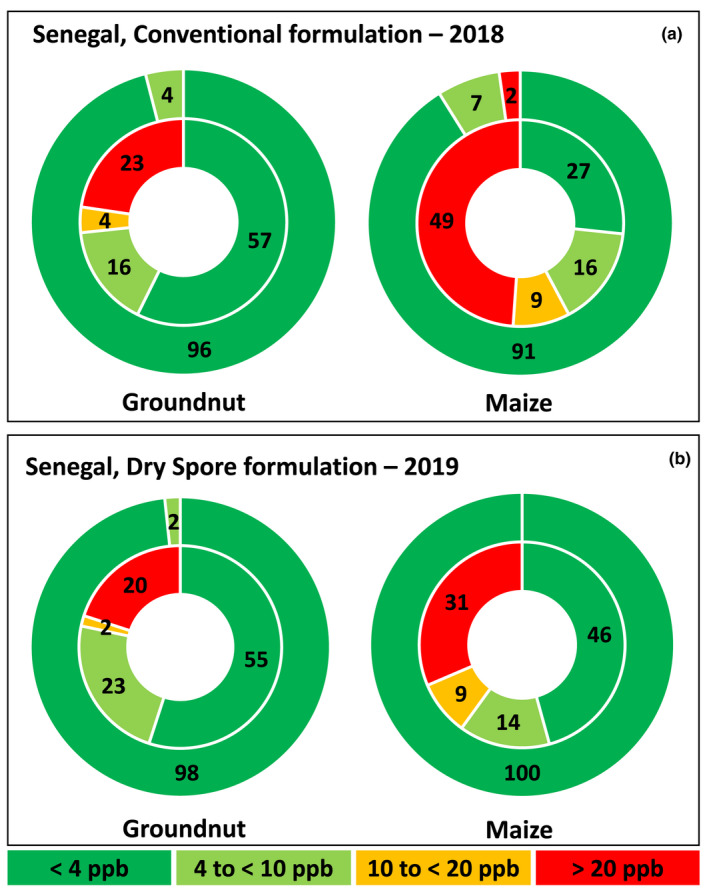
Trials conducted in six regions in Senegal with conventional formulation in 2018 (A) and dry spore formulation in 2019 (B). The percentage of groundnut and maize crops in each of four total aflatoxin concentration categories is indicated by different colours. The outer circle shows Aflasafe SN01‐treated crops while the inner circle shows untreated crops. For groundnut, there were 150 and 120 farmer field trials in 2018 and 2019, respectively. For maize, there were 90 and 70 farmer field trials in 2018 and 2019, respectively. In all cases, half of the trials were treated and the rest untreated.

Although promising results were obtained in the laboratory in Ibadan and Kahone, the true value of the dry spore product was revealed when tested in commercial fields paired with untreated fields. Moreover, it was necessary to compare the innovation’s performance with that of biocontrol product manufactured in the conventional manner (manufactured the previous year, 2018, in Ibadan and sent via sea freight to Senegal). Field evaluations revealed that both products were equally effective in limiting aflatoxin contamination. Groundnut treated with either product had very low total aflatoxin levels (at or below 2 ppb) while in nearby crops the aflatoxin levels were in many cases extremely unsafe (Table 2).

Despite being unpaired comparisons and conducted in different years, crops treated in The Gambia with conventional product in 2018 by NFSPMC farmers (Senghor *et al*., 2021; Table 3) and with dry spore product in 2019 by NFSPMC and FAO farmers (Table 3) harboured very low aflatoxin levels. In Senegal, crops treated in 2018 by BAMTAARE farmers (conventional product) and in 2019 by BAMTAARE and ASPRODEB farmers (dry spore product) had safe aflatoxin levels (Fig. 3; Table S1). In contrast, untreated crops in each of the two years, in both countries, contained in most cases, on an average, dangerous aflatoxin content.

Production of several biopesticides having as active ingredient spores of a fungus is challenged by difficulties to mass‐produce and harvest the spores, appropriate formulation procedures, and formulated product stability (Bateman, 2004; Jaronski and Mascarin, 2013). Viability of the spores may be reduced or lost during harvesting and/or manufacturing. For example, the viability of dry spores of *Trichoderma harzianum*, which controls several plant pathogens, is highly dependent on the drying and dehydration temperature as well as the conditions in which the spores are stored (Fernández‐Sandoval *et al*., 2012). Spore viability of several fungi is also dictated by the type and concentrations of additives and protectants applied to the spores (Harman *et al*., 1991; Stephan and Zimmerman, 1998). On the other hand, mass production, harvesting and processing of spores of atoxigenic isolates of *A. flavus* is relatively simple to achieve (Senghor *et al*., 2020). In addition, aflatoxin biocontrol products remain stable for well over 2 years (unpublished results). In the current study, additives or protectants were not incorporated into the dry spores during storage and shipment; the spores were kept solely as a mass of dry powder. Part of the results of the current study demonstrates that *Aspergillus* spores subjected to industrial processes are not fragile as spores of many other fungi. Indeed, several studies report that *Aspergillus* spores are sturdy. For example, viability of spores of another *Aspergillus* species, *A. niger*, is minimally affected when subjected to severe vacuuming conditions and high temperatures (Davis *et al*., 1963) and even to high levels of radiation (Cortesão *et al*., 2020).

Prior to industrial manufacturing of aflatoxin biocontrol products in Africa, the laboratory‐scale archetype process consisted in inoculating sterile sorghum grain with spore suspensions of the active ingredient fungi, incubating the grain at 31°C for 18 h to allow internal fungal growth, and drying the inoculated grain for 4 days at 55°C (Atehnkeng *et al*., 2014). Viability of fungi composing products manufactured in that manner was not affected by the drying process and the products were highly effective in limiting aflatoxin contamination (Bandyopadhyay *et al*., 2016, 2019) – although their production was not cost‐effective. Therefore, it was not surprising to find that both the relatively gentle vacuuming conditions and the drying step (37°C, 2 days) did not affect the viability of the spores as demonstrated by the field effectiveness of the dry spore product (Tables 2 and 3; Fig. 3). Reasons for superior resilience of *Aspergillus* spores compared to those of other fungi used in biocontrol formulations deserve further investigations.

Cole and Dorner (2001) patented a method to produce aflatoxin biocontrol products by coating grain with dry spores of atoxigenic fungi diluted in a vegetable oil and then adding a siliceous material in a separate step ‘to form a free‐flowing formulation which can be directly applied to soil’. The method used in Kahone is different. The spores were diluted in water and then mixed with a polymer and blue dye. This master suspension was then coated in a single step using a seed treater. Materials to aid in the performance of the dry product were not added. The blue dye was added to differentiate the product from regular sorghum.

The aflatoxin biocontrol technology, developed for use in the United States (Cotty *et al*., 2007), was adapted and improved for use in SSA. In the United States, critical work was conducted to determine best formulations and substrates for atoxigenic isolates to perform effectively in the field and outcompete toxigenic fungi (Cole and Dorner, 2001; Dorner, 2004; Cotty *et al*., 2007; Jaime‐Garcia and Cotty, 2008; Jaime *et al*., 2014). Efforts described in the current study are part of ongoing actions to further improve the archetype of the technology, making it more versatile and easier for manufacturers to produce the biocontrol product, and less expensive to smallholder farmers without compromising its effectiveness. The laboratory‐scale process to dry the spores, however, needs further improvement to make it more cost‐efficient. Some instruments allow harvesting spores using dry processes to produce commercial biopesticides (Bateman, 2004). A highly effective biopesticide produced with spores harvested in a dry manner is Green Muscle® that has a *Metarhizium anisopliae* var. *acridum* isolate as active ingredient for the control of grasshoppers and locusts (Douthwaite *et al*., 2001; Schreurs *et al*., 2019). Directly dislodging and concentrating spores from fermentation medium using an industrial spore harvester would eliminate the need to harvest spores from suspension and the drying step. This will result in faster, cheaper production of the spores of the active ingredient isolates.

Manufacturers procuring dry spores do not need to construct a laboratory to produce fresh spores. Opting for the dry spore innovation results in reduced construction, capital investment, and running costs, thus making the biocontrol product cheaper for farmers. Moreover, availability of dry spores, which have a shelf‐life of at least 1 year (unpublished results), will reduce the lead time to manufacture the product once clients confirm their order. The reduced volume of the active ingredients is an advantage to manufacturers since the spores can be procured and stored in a refrigerator until use. In addition, high spore quality and reduced risk of product contamination can result from using dry spores. Manufacturers in Nigeria and Tanzania have decided to use dry spores produced by IITA (a non‐profit institution) at cost through a public–private partnership.

Contamination events in aflatoxin‐prone areas are increasing in both numbers and severities. Farmers rely on rapid development and deployment of effective, affordable tools to produce safe crops. The actions reported in this work are examples of how relatively frugal, but well envisioned and coordinated efforts can overcome infrastructure limitations to make implementation of aflatoxin management a reality. A much‐needed innovation was rapidly conceptualized, developed, tested, and validated in the laboratory, and used industrially to produce a reliable field aflatoxin mitigation tool. Thousands of commercial farmers in Senegal and The Gambia had access to the tool and used it as a part of an integrated aflatoxin management programme that included pre‐ and post‐harvest interventions. Most farmers using the integrated management programme were able to produce crops with safe aflatoxin content.

Delays in availability of an aflatoxin biocontrol product and failure to implement proper aflatoxin management can cause unnecessary aflatoxin exposure, which is high in both Senegal and The Gambia (Kuniholm *et al*., 2008; Watson *et al*., 2015). At harvest, aflatoxin levels in untreated crops reached up to 170 ppb in 2018 and up to 380 ppb in 2019. Those levels can increase under sub‐optimal storage (Senghor *et al*., 2020). Only a small fraction of the untreated crops met the stringent EU standards and a large proportion of the untreated crops had insufficient quality to be considered safe for human consumption (Tables 2 and 3, Fig. 3). With an available manufacturing facility in Kahone with capacity to produce 10 tons per day of Aflasafe SN01 for use in Senegal, The Gambia, and Mali, food safety and farmers’ income in the region are expected to significantly improve. Of course, such improvements are highly dependent on use of an integrated aflatoxin management system converging necessary technologies, policies, and health interventions to significantly reduce aflatoxin contamination throughout the value chain.

## Experimental procedures

### Fungi constituting the aflatoxin biocontrol product Aflasafe SN01

The biocontrol product Aflasafe SN01 contains as active ingredient the atoxigenic *A. flavus* isolates SS19‐14, MS14‐19, M2‐7 and M21‐11. These isolates belong to AAV‐SS19‐14, AAV‐MS14‐19, AAV‐M2‐7, and AAV‐M21‐11, respectively. The four AAVs have been detected across Senegal (Diedhiou *et al*., 2011; Senghor *et al*., 2020) and The Gambia (Senghor *et al*., 2021). The product effectively reduces aflatoxin contamination of maize and groundnut in both Senegal (Senghor *et al*., 2020) and The Gambia (Senghor *et al*., 2021).

### Production of Aflasafe SN01 active ingredient

To prepare the active ingredient of Aflasafe SN01, working cultures were made from mother cultures (maintained at IITA‐Ibadan in silica grains, for long‐term storage) of each of the four atoxigenic AAVs constituting the product. Each isolate was grown on 5–2 agar [5% V8 Juice (Campbell Soup Company, Camden, NJ, USA), 2% Bacto‐agar (Difco Laboratories Inc., Detroit, MI, USA), pH 6.0)] for 5 days at 31°C. Spores were dislodged and suspended in 0.1% TWEEN®80 and adjusted to 10^6^ spores ml^‐1^ using a turbidimeter (Atehnkeng *et al*., 2014).

Production of billions of spores is possible when independently inoculating each atoxigenic isolate on several bottles containing autoclaved sorghum grain (Agbetiameh *et al*., 2019). In the current study, each atoxigenic isolate was inoculated in six 250‐ml glass bottles containing sorghum grain previously pre‐conditioned to 30% moisture content. The pre‐conditioning was done in sterile 1‐l plastic bottles rolled for 4 h on a 240 Vac Benchtop Roller (Wheaton, Millville, NJ, USA). Thereafter, 30 g of pre‐conditioned grain were added to the glass bottles along with two Teflon balls (1/2” diameter) and autoclaved (20 min, 121°C). Once cooled, each bottle was independently inoculated with 4 ml spore suspension (5 × 10^5^ spores ml^‐1^) of an atoxigenic isolate. To allow sterile aeration, each bottle was covered with sterile Tyvek® membrane (7 cm^2^) secured with a sterile polypropylene pour ring (GL 45; DWK Life Sciences, Rockwood, TN, USA). The membrane allows gas exchange, but its pores are not large enough to provide access or escape of microorganisms. All bottles were incubated for 7 days (35°C, dark). There were six bottles per isolate (24 bottles in total) which yield enough spores to manufacture six tons of biocontrol product.

### Harvesting and drying of atoxigenic *A. flavus* isolates

After incubation, all bottles were transferred to a biosafety level 2 cabinet. Tyvek membranes were aseptically removed and 125 ml sterile 0.03% TWEEN®20 was added to each bottle. Then, bottles were covered with sterile plastic caps, tightly closed, and placed on a Roto‐Shake Genie reciprocal shaker (Scientific Industries, Bohemia, NY, USA) to dislodge spores (200 rpm, 20 min). The Teflon balls aided dislodging spores from sorghum grains (Agbetiameh *et al*., 2019). Sterile funnels holding sterile sieves (1.7 mm^2^; Newark Wire Cloth Co., Clifton, NJ, USA) were used to separate grains from the suspension (Fig. 1A). Dislodged spores in suspension were transferred to sterile 250 ml glass bottles and tightly covered with sterile caps (Fig. 1B). These spore suspensions were either appropriately diluted and termed as freshly produced spores or further processed to produce dry spores.

Spores were separated from liquid using a vacuum pump (1 stage Vacuum Pump, VE 125; Nanjing T‐Bota Scietech Instrument and Equipment Co. Ltd., Nanjing, China) attached to 500 ml filtration flasks. A single piece of sterile Tyvek membrane was cut to fit sterile vacuum funnels (Fig. 1C). Suspensions were slowly poured in the centre of the membrane. After filtration, spores remained on the membrane as a crust and were almost completely dry. Using sterile spatulas, the spore crusts were transferred to sterile, labelled 40 ml glass vials (Fig. 1D). Vials were covered with sterile Tyvek membrane (5 cm^2^) secured with a rubber band disinfected with 70% ethanol, wrapped in foil paper, and dried in a forced‐air oven (37°C, 48 h). After drying, vials containing the dry spores were stored at room temperature (~25°C) until used in the evaluations.

### Assessment of viability of dry spores and freshly produced spores

The viability of dry spores was evaluated after 2, 5, 10, 15, and 30 days of storage. In each evaluation, freshly harvested spores were used as control to determine if the drying process and storage period influenced spore viability (spore germination) and/or allowed microbial contamination of dry spores. From each vial containing dry spores, 0.3 g were aseptically transferred to sterile 40 ml glass vials containing 29.7 ml sterile distilled water. The suspension (3 × 10^9^ spores ml^‐1^) was vortexed and 600 µl aliquots were uniformly spread in triplicates on both water agar (WA; 2% Bacto‐agar) and violet red bile agar (VRBA; Difco Laboratories Inc., 41.5 g l^‐1^, pH 7.4). For fresh spores (harvested ~ 20 min prior to plating), suspensions containing the same concentration as above were plated. All plates were incubated at 31°C for 2 days.

### Biocontrol product preparation using dry spores and freshly harvested spores

The same spore suspensions used to assess spore viability of dry spores and freshly harvested spores were used to manually prepare biocontrol products using a laboratory‐scale process. Briefly, for both dry spores and freshly harvested spores, the suspensions of each atoxigenic isolate were adjusted to 4 × 10^7^ spores ml^‐1^ and used to prepare experimental products containing single isolates. For each isolate, 10 ml adjusted spore suspension was mixed with 10.5 ml sterile distilled water, 1.5 ml polymer (Sentry™, Precision Laboratories, Waukegan, IL, USA), and 2 ml blue dye (Prism™, Milliken & Company, Spartanburg, SC, USA) to coat 1 kg of sterile roasted sorghum grain. Coating was performed by manually shaking the required quantity of sterile sorghum grain and spore–polymer–dye suspension in a container until the grains were uniformly blue in colour. All manually produced products were subjected to standard quality tests as described previously (Agbetiameh *et al*., 2019; Senghor *et al*., 2020). These tests determined (i) proportions of germinated grains, (ii) grains with *A. flavus* growth, (iii) grains with other microbial (fungi or bacteria) growth, (iv) number of spores produced per g of product, and (v) identity of the *A. flavus* fungi recovered from the grains.

### Production of dry spores sent to Kahone, Senegal

Dry spores enough to manufacture 200 tons of Aflasafe SN01 were produced in Ibadan as described above. In total, 200 bottles containing sterile pre‐conditioned sorghum grain were inoculated per atoxigenic isolate. Two batches were produced, 100 bottles per isolate per batch, on 30 April 2019 and 3 June 2019. In all, 4 kg of dry spores were produced and sent to Dakar via air freight under appropriate export permit from the Nigeria Agricultural Quarantine Service and import permit from La Direction de Protection Végétaux (DPV) of Senegal. The dry spores were placed in sterile 40 ml glass vials tightly closed and sent within sealed cardboard boxes. After transport via road at ambient temperature from Dakar to BAMTAARE plant in Kahone (~3 h drive), the dry spores were kept at 4°C until used to manufacture the product during July and August 2019.

### Manufacturing and quality control of Aflasafe SN01 produced in Kahone using dry spores

In Kahone, 5 g dry spores of each atoxigenic isolate (20 g total) were combined with 10 l sterile distilled water (resulting in 4 × 10^7^ spores ml^‐1^) to prepare 1 ton of Aflasafe SN01 product (Fig. 2). The suspension was placed in polypropylene carboys (VWR, Radnor, PA, USA), combined with 10.5 l sterile water, 1.5 l polymer and 2 l blue dye, agitated for proper mixing, and used to coat roasted, sterile sorghum grain with a seed treater (Fig. 2). Quality tests for all batches (*n* = 360; two per ton) of product industrially manufactured with dry spores were conducted in Kahone as previously described (Agbetiameh *et al*., 2019; Senghor *et al*., 2020).

### Paired field evaluation of biocontrol products manufactured with dry spores and conventionally

Commercial usage of Aflasafe SN01 in Senegal and The Gambia by smallholder farmers started in 2016. Before constructing its manufacturing facility, BAMTAARE procured the conventionally manufactured product from the IITA‐Ibadan plant (Bandyopadhyay *et al*., 2016; Senghor *et al*., 2020). BAMTAREE procured 20, 50, and 200 tons of product in 2016, 2017, and 2018, respectively, for use in Senegal and The Gambia. Part of the product manufactured in 2018 (conventional product) was used in 2019 in The Gambia. The performance of Aflasafe SN01 manufactured in 2018 and used in 2019 was compared with that of product manufactured with dry spores and also used in 2019 (Table 2).

Farmers belonging to The National Food Security, Processing and Marketing Corporation (NFSPMC) of The Gambia participated in the commercial comparison trials. NFSPMC farmers have used the biocontrol product since the testing phase (2014–2015) and due to periodic training and usage are familiar with the application method and practices to maximize the product’s effectiveness (Senghor *et al*., 2020). The trials were conducted in the Central River and North Bank regions, with 20 treated fields per region (i.e. 10 with conventional product from 2018 and 10 with dry spore product from 2019).

Farmers grew their groundnut crops following recommendations given by NFSPMC. Farmers planted either 28–206 [a late‐maturity variety; 125 days growing cycle; flowering time ~ 45 days after planting (dap)] or 73–33 (an intermediate‐maturing variety; 115 days growing cycle; flowering time ~ 40 dap) two of the most common groundnut varieties in The Gambia (ICRISAT, 1990). In general, groundnut crops were planted after the first rains during the first week of July. Farmers weeded their fields by hand and animal‐drawn hoe, top‐dressed with a complex fertilizer, and earthed‐up (i.e. piling up soil around the base of the plants) before application of the biocontrol product to avoid burying it. The products were broadcasted by hand at a rate of 10 kg ha^‐1^ at 30‐to‐35 dap. For each treated field, a neighbouring field–managed by the same farmer and planted with the same variety–at least 200 m apart was selected as the corresponding untreated field. Field sizes ranged from 0.5 to 2 ha. Production of groundnut in all fields was dependent on rainfalls, which were erratic.

### Field evaluations in Senegal and The Gambia of biocontrol products manufactured with the conventional method (2018) and with dry spores (2019)

Although not paired comparisons, and conducted in different years, biocontrol effectiveness was also monitored in Senegal and The Gambia. In The Gambia, conventional product was used by NFSPMC farmers in 2018 while dry spore product was used in 2019 by both NFSPMC farmers and farmers participating in a Food and Agriculture Organization (FAO) scheme. All of these were groundnut trials conducted in multiple fields (either 10 or 15 treated fields) of multiple regions (Table 3). Each treated field had an accompanying untreated field as above. The results of the 2018 trials by NFSPMC farmers have been reported (Senghor *et al*., 2021).

In Senegal in 2018, conventional product was used in both maize and groundnut fields of farmers working with BAMTAARE in five regions (Table S1). There were 15 treated fields per region and each field had an accompanying untreated field as above. In 2019, farmers working either with BAMTAARE or the Association Sénégalaise pour la Promotion du Développement par la Base (ASPRODEB) treated maize and/or groundnut fields with dry spore product (Table S1). There were 10–30 treated fields in each of the four regions and each treated field had an accompanying untreated field.

Groundnut varieties, planting time and agronomic practices were similar as those described above for The Gambia. For maize, farmers planted diverse improved or landrace varieties that flowered around 60 to 70 dap. Maize was also planted during the first week of July after the first rains. Farmers weeded their maize by hand and animal‐drawn hoe, top‐dressed with urea, and earthed‐up before broadcasting the biocontrol product by hand 2‐to‐3 weeks before flowering. For each treated maize field, a neighbouring untreated maize field was maintained in a similar manner as groundnut. Field sizes ranged from 0.5 to 5 ha. Crop production in all fields was dependent on rainfalls, which were erratic.

### Crop sampling and aflatoxin quantification

Regardless of treatment, year, and country, crops were harvested at physiological maturity. In all trials, extension agents collected representative grain samples (~2 kg) from each field. Aflatoxin quantification in The Gambia was conducted at NFSPMC and FAO premises by NFSPMC technicians. In Senegal, aflatoxin quantification was done at BAMTAARE and ASPRODEB premises by BAMTAARE technicians.

In both countries, some farmers participated in the aflatoxin testing process whereas others were observers. GIPSA‐approved Neogen Reveal® Q + for Aflatoxin kits (Neogen Corp., Lansing, MI, USA) were used. Briefly, grains were milled using a laboratory blender (Waring Commercial, Springfield, MO, USA) for 30 sec in a 1‐l stainless steel blending jar (MC‐2). The jar was thoroughly washed with 80% ethanol to prevent aflatoxin cross contamination between samples. For each sample, a 20‐g sub‐sample was transferred into a 250 ml glass bottle and 100 ml 65% ethanol was added. The mixture was shaken by hand (3 min), allowed to settle (3 min) and filtered through Whatman No. 1 filter paper (Whatman Intl. Ltd., Maidstone, UK) into a Tri‐Pour® beaker. Thereafter, 500 µl sample diluent was transferred to a sample cup and 100 µl sample filtrate was added. A 100 µl aliquot of diluted sample was transferred into a new cup and mixed thoroughly by aspiration. A lateral flow strip was placed in the sample cup for 6 min and then the strip was read on a Neogene AccuScan Gold Reader. The limit of detection was 1 part per billion (ppb) total aflatoxin.

### Data analyses

Data were summarized and analysed using SAS v9.4 (SAS Institute Inc., Cary, NC, USA). Data for all dependent variables (CFU/g of dry spores, CFU/grain of formulated product and aflatoxin levels in treated and untreated crops) were log‐transformed, using the equation [*y* = log(response variable + 1)] to homogenize the variance prior to analysis. CFU means between dry spores and freshly harvested spores, and in aflatoxin content means between treated and untreated crops in both The Gambia and Senegal were compared with Student’s *t*‐test (α = 0.05) using the TTEST procedure of SAS.

CFU/grain data were subjected to a factorial ANOVA to determine the differences among Dry and Fresh spores, isolates, storage period, and their interactions. The treatment means were compared according to Tukey HSD test (α = 0.05).

For the field comparison of the two formulations in The Gambia in 2019, Conventional and Dry Spore, the Kruskal–Wallis one‐way nonparametric test was performed and total aflatoxin means were compared at α = 0.05. For the field comparison in The Gambia conducted in different years, data were subjected to a factorial ANOVA to determine the differences among regions, farmers’ organizations, year, formulation, treatment, and their interactions. The treatment means were compared according to Tukey HSD test (α = 0.05).

## Conflict of interest

The authors receive no direct financial benefit from the manufacturing and marketing of aflatoxin biocontrol products mentioned in this article. The Aflasafe name is a Trademark of the International Institute of Tropical Agriculture (IITA). IITA used to manufacture Aflasafe for use in Nigeria, Senegal, Kenya, Burkina Faso, The Gambia, and Ghana. Manufacturing and distribution responsibilities have been licensed to private or public sector entities in a few African countries. IITA charges a small licensing fee to manufacturers for use of the Aflasafe name and cost associated with technology transfer and technical backstopping. A. Ortega‐Beltran, L. Kaptoge, A.L. Senghor, M.O.S. Aikore, H. Momanyi, M. Konlambigue, T.D.O. Falade, and R. Bandyopadhyay are employed by IITA.

## Supporting information


**Table S1**. Aflatoxin content in groundnut and maize sampled from (i) fields treated with the biocontrol product Aflasafe SN01 formulated either conventionally (2018) or with dry spores (2019) and (ii) untreated fields in six regions of Senegal.

## Data Availability

The data that support the findings of this study are available from the corresponding author upon reasonable request.
