# Supplementary material for: Can it be all more simple? Manufacturing aflatoxin biocontrol products using dry spores of atoxigenic isolates of Aspergillus flavus as active ingredients
Source: Microb Biotechnol. 2021 Mar 23;15(3):901–14. doi: 10.1111/1751-7915.13802 (PMC8913866; doi:10.1111/1751-7915.13802)
Supplement: Supplementary file 1 — Table S1. Aflatoxin content in groundnut and maize sampled from (i) fields treated with the biocontrol product Aflasafe SN01 formulated either conventionally (2018) or with dry spores (2019) and (ii) untreated fields in six regions of Senegal. [file MBT2-15-901-s001.docx]

**Supplementary Table 1.** Aflatoxin content in groundnut and maize sampled from i) fields treated with the biocontrol product Aflasafe SN01 formulated either conventionally (2018) or with dry spores (2019) and ii) untreated fields in six regions of Senegal.

| Region | Crop | Farmers organization ^a^ | Year | Formulation ^b^ | Treatment ^c^ | n | Total aflatoxin ppb ^d^ | | | | Red (%) ^f^ |
| --- | --- | --- | --- | --- | --- | --- | --- | --- | --- | --- | --- |
|  |  |  |  |  |  |  | Min | Max | Average ^e^ | Variance |  |
| Kaolack | Groundnut | BAMTAARE | 2018 | Conventional | Treated | 15 | 2.0 | 4.1 | 2.4* | 0.3 | 55.1 |
|  |  |  |  |  | Untreated | 15 | 2.1 | 18.2 | 5.3 | 19.0 |  |
|  |  |  |  |  |  |  |  |  |  |  |  |
| Koungheul | Groundnut | BAMTAARE | 2018 | Conventional | Treated | 15 | 2.0 | 3.6 | 2.3** | 0.2 | 86.5 |
|  |  |  |  |  | Untreated | 15 | 2.2 | 133.0 | 17.1 | 1,242.8 |  |
|  |  |  |  |  |  |  |  |  |  |  |  |
|  | Maize | BAMTAARE | 2018 | Conventional | Treated | 15 | 2.0 | 4.9 | 2.4*** | 0.5 | 93.6 |
|  |  |  |  |  | Untreated | 15 | 2.0 | 104.7 | 37.8 | 1,317.5 |  |
|  |  |  |  |  |  |  |  |  |  |  |  |
| Kolda | Groundnut | BAMTAARE | 2018 | Conventional | Treated | 15 | 2.0 | 4.7 | 2.8** | 0.5 | 93.1 |
|  |  |  |  |  | Untreated | 15 | 3.2 | 150.0 | 51.3 | 2,870.8 |  |
|  |  |  |  |  |  |  |  |  |  |  |  |
|  | Maize | BAMTAARE | 2019 | Dry spores | Treated | 10 | 2.0 | 2.9 | 2.2 | 0.1 | 15.4 |
|  |  |  |  |  | Untreated | 10 | 2.0 | 5.5 | 2.5 | 1.3 |  |
|  |  |  |  |  |  |  |  |  |  |  |  |
| Tambacounda | Groundnut | BAMTAARE | 2018 | Conventional | Treated | 15 | 2.0 | 4.3 | 2.2* | 0.4 | 85.0 |
|  |  |  |  |  | Untreated | 15 | 2.0 | 106.5 | 14.8 | 1,012.9 |  |
|  |  |  |  |  |  |  |  |  |  |  |  |
|  | Groundnut | BAMTAARE | 2019 | Dry spores | Treated | 30 | 2.0 | 8.6 | 2.3*** | 1.5 | 91.9 |
|  |  |  |  |  | Untreated | 30 | 2.0 | 150.0 | 28.6 | 2,403.8 |  |
|  |  |  |  |  |  |  |  |  |  |  |  |
|  | Groundnut | ASPRODEB | 2019 | Dry spores | Treated | 15 | 2.0 | 2.0 | 2.0 | 0.0 | 59.1 |
|  |  |  |  |  | Untreated | 15 | 2.0 | 36.9 | 4.9 | 80.3 |  |
|  |  |  |  |  |  |  |  |  |  |  |  |
|  | Maize | BAMTAARE | 2018 | Conventional | Treated | 15 | 2.0 | 23.6 | 3.7** | 30.5 | 88.7 |
|  |  |  |  |  | Untreated | 15 | 2.0 | 118.5 | 32.7 | 1,740.5 |  |
|  |  |  |  |  |  |  |  |  |  |  |  |
|  | Maize | BAMTAARE | 2019 | Dry spores | Treated | 10 | 2.0 | 3.7 | 3.0* | 0.4 | 81.1 |
|  |  |  |  |  | Untreated | 10 | 3.2 | 46.7 | 15.7 | 288.9 |  |
|  |  |  |  |  |  |  |  |  |  |  |  |
| Velingara | Groundnut | BAMTAARE | 2018 | Conventional | Treated | 15 | ND | 3.8 | 2.2** | 0.7 | 84.0 |
|  |  |  |  |  | Untreated | 15 | 2.0 | 75.9 | 14.0 | 498.7 |  |
|  |  |  |  |  |  |  |  |  |  |  |  |
|  | Groundnut | ASPRODEB | 2019 | Dry spores | Treated | 15 | 2.0 | 2.0 | 2.0* | 0.0 | 89.7 |
|  |  |  |  |  | Untreated | 15 | 2.0 | 84.0 | 19.5 | 808.9 |  |
|  |  |  |  |  |  |  |  |  |  |  |  |
|  | Maize | BAMTAARE | 2018 | Conventional | Treated | 15 | 2.0 | 4.8 | 2.8** | 0.8 | 92.8 |
|  |  |  |  |  | Untreated | 15 | 2.1 | 144.0 | 38.5 | 2,412.6 |  |
|  |  |  |  |  |  |  |  |  |  |  |  |
| Koupentome | Maize | BAMTAARE | 2019 | Dry spores | Treated | 15 | 2.0 | 2.0 | 2.0** | 0.0 | 96.1 |
|  |  |  |  |  | Untreated | 15 | 2.0 | 150.0 | 51.2 | 3,402.3 |  |

^a^ Farmers working with BAMTAARE or the Association Sénégalaise pour la Promotion du Développement par la Base (ASPRODEB).

^b^ Dry spore refers to product manufactured in Kahone, Senegal, in 2019 with a new process using dry spores produced in Ibadan, Nigeria. Conventional refers to product manufactured in Ibadan during 2018 using the standard process previously described (Bandyopadhyay et al., 2016).

^c^ Treated refers to fields to which Aflasafe SN01, regardless of formulation, was applied at the rate of 10 kg/ha. Untreated were nearby fields separated by at least 200 m from corresponding treated field in which no biocontrol product was applied.

^d^ Aflatoxin values are in parts per billion (ppb).

^e^ Means of aflatoxin values were compared independently between treated and untreated crops in each district and each year. Treated values with one, two, or three asterisks (*) significantly differed from corresponding untreated values by Student’s *t*-test (α = 0.05, 0.001, and 0.0001 respectively).

^f^ Percentage reduction was calculated as follows: ([mean of untreated − mean of Aflasafe SN01 treated]/ mean of untreated) × 100.
